# HIV-infected patients rarely develop invasive fungal diseases under good immune reconstitution after ART regardless high prevalence of pathogenic filamentous fungi carriage in nasopharynx/oropharynx

**DOI:** 10.3389/fmicb.2022.968532

**Published:** 2022-11-02

**Authors:** Xiaoman Chen, Yi Cao, Meijun Chen, Haodi Wang, Peishan Du, Hong Li, Huolin Zhong, Quanmin Li, Santao Zhao, Zhenjiang Yao, Wanshan Chen, Weiping Cai, Xiaoping Tang, Linghua Li

**Affiliations:** ^1^Infectious Disease Center, Guangzhou Eighth People’s Hospital, Guangzhou Medical University, Guangzhou, China; ^2^Department of Epidemiology and Health Statistics, School of Public Health, Guangdong Pharmaceutical University, Guangzhou, China

**Keywords:** human immunodeficiency virus, pathogenic filamentous fungi, filamentous fungi, invasive fungal disease, immune reconstitution, carrier, smoking, opportunistic infections

## Abstract

**Purpose:**

We aimed to investigate the prevalence and risk factors of filamentous fungi (FF) carriage in human immunodeficiency virus (HIV)-infected patients in Guangdong province, along with its subsequent incidence of invasive fungal disease (IFD).

**Methods:**

Seven hundred and sixteen HIV-infected individuals from the outpatient clinic and 293 sex-matched healthy controls were recruited prospectively from May 1 to August 31, 2017. Fungi were isolated from oropharyngeal and nasopharyngeal swabs, then identified by morphological and molecular biological techniques. Logistic regression analysis was used to identify risk factors of pathogenic FF carriage. Pathogenic FF carriers were followed up through the end of 2019.

**Results:**

Of the 716 included HIV-infected patients, 602 (84.1%) were male, the median age was 34 (27–42) years, and the median CD4+ count was 385 (254–542) cells/μl. Pathogenic FF were isolated in 119 (16.6%) cases with HIV infection and 40 (13.7%) healthy controls. Mucorales were found in 3 HIV-infected individuals and *Talaromyces marneffei* in 2 HIV-infected individuals, but not in healthy controls. History of cured opportunistic infections (OIs; OR, 1.97; 95% CI, 1.23–3.13, *p* = 0.004), and smoking (OR, 1.55; 95%CI, 1.03–2.32, *p* = 0.035) were independent risk factors of pathogenic FF carriage in HIV-infected individuals. A total of 119 pathogenic FF carriers with HIV infection were followed. During follow-up, 119 (100%) cases received antiretroviral therapy (ART) for at least 28 months, 107 (90%) cases had CD4+ counts>200 cells/μl, and none developed IFD.

**Discussion:**

Pathogenic FF carriage is common in HIV-infected individuals but may not develop IFD in those who achieved immune reconstitution. Smoking and cured OIs history increase the risk of pathogenic FF carriage. Smoking abstinence and ART adherence are especially important for these patients.

## Introduction

Invasive fungal disease (IFD) is an opportunistic infection (OI) in immunocompromised patients, with a high mortality rate of more than 50% despite the availability of antifungal drugs (Brown et al., 2012). Individuals with human immunodeficiency virus (HIV) infection are a high-risk population for IFD. It is estimated that up to 1 million patients with HIV infection suffered from IFDs annually, with about 500,000 of them dying each year (Armstrong-James et al., 2014; Chastain et al., 2017). Pathogenic filamentous fungi (FF) have become common in HIV-infected individuals with IFD, mainly including *Talaromyces marneffei*, pathogenic *Aspergillus*, and Mucorales (Marukutira et al., 2014; Moreira et al., 2016; CAO et al., 2021). In Southern China, the prevalence rate of talaromycosis in HIV-positive inpatients was up to 16.0%, with a mortality rate of 50.6% without timely antifungal treatment (Hu et al., 2013; Ying et al., 2020). Aspergillosis occurred in 4.4% of HIV-infected patients with IFD, and the survival possibility was lower than cryptococcosis (Marukutira et al., 2014). Mucormycosis is rare in HIV-infected individuals but may occur in patients with advanced acquired immune deficiency syndrome (AIDS), with a reported mortality rate of 52.2% (Moreira et al., 2016).

IFD in HIV-infected patients has an insidious onset, expresses progress rapidly, and carries a poor prognosis. Early screening, diagnosis, and treatment of IFD are crucial to reducing IFD-related mortality in HIV-infected patients. The nasopharynx and oropharynx are the initial points of entry to the digestive and respiratory tract, colonizing with a wide variety of fungi. Previous studies have focused on *Candida* (Maurya et al., 2013; Hall and Noverr, 2017; Goulart et al., 2018; Aboualigalehdari et al., 2020). *Candida* colonizes about 40–50% of HIV-infected patients and is associated with invasive candidiasis (Maurya et al., 2013; Goulart et al., 2018; Von Lilienfeld-Toal et al., 2019; Aboualigalehdari et al., 2020). However, few studies have explored the asymptomatic carriage of pathogenic FF, such as *Talaromyces marneffei*, pathogenic *Aspergillus*, and Mucorales. This study aimed to investigate the prevalence of asymptomatic FF carriage in nasopharynx and oropharynx of HIV-infected individuals in Guangdong province, and follow their incidence of IFD, thus providing a theoretical basis for the prevention and surveillance of IFD in HIV-infected patients.

## Materials and methods

### Study design

This was an observational study with participants recruited prospectively, including two parts: (1) A cross-sectional study to investigate the prevalence of fungal carriage in HIV-infected individuals and healthy populations, and risk factors of pathogenic FF carriage in HIV-infected patients; (2) A follow-up study to investigate the occurrence of IFD among pathogenic FF carriers with HIV infection. We randomly enrolled HIV-infected individuals from the outpatient clinic at the Department of Infectious Diseases of the Guangzhou Eighth People’s Hospital (GEPH) from May 1, 2017 to August 31, 2017. Meanwhile, healthy controls were recruited from a physical examination center in Guangzhou. All participants were adults ≥18 years of age at enrolment. The exclusion criteria were as follows: (1) the presence of oral fungal infection by clinical examination; (2) received antibiotic or antifungal treatment within the past 2 weeks; (3) received corticosteroids within the past 28 days, or immunosuppressants within the past 3 months; (4) suffered from acute infection at enrolment; (5) skin or mucosa lesions; (6) suffered from OIs, severe underlying or systemic diseases, or tumor at enrolment; (7) pregnancy or lactation. Ethical approval was provided by the Medical Ethics Committee of the GEPH (Approval No. 20150155). All patients signed an informed consent form.

HIV infection was confirmed by positive HIV enzyme-linked immunosorbent assay (ELISA) and a confirmatory Western blot (WB), or RNA Nucleic Acid Amplification Test (NAAT) testing. All participants underwent medical history questionnaires at enrolment. Oropharyngeal and nasopharyngeal swabs were collected at enrolment for fungal culture and strain identification. Risk factors of pathogenic FF carriage were explored. Subsequently, pathogenic FF carriers with HIV infection were followed through the end of 2019 until their first diagnosis of IFD, or their last follow-up.

### Data collection

All participants underwent medical history questionnaires at enrolment. The following data were collected. Demographic data: age and gender; Exposure history within 6 months: rodent exposure, hay or chaff exposure; Lifestyle habits: smoking history, alcoholism; Past medical history within 1 year: cured respiratory tract infections, cured OIs, other infectious diseases, and medication history; HIV-related data: history of antiretroviral therapy (ART). Plasma HIV RNA (viral load) and CD4+ counts were collected from the patient’s electronic medical records.

Alcoholism was defined in this study as follows: (1) alcohol abuse for more than 5 years and ethanol consumption ≥40 g/d for men and ≥ 20 g/d for women, or (2) heavy drinking in the most recent 2 weeks equivalent to ethanol ≥80 g/d; OIs were defined as infections caused by conditional pathogens not harmful to healthy populations but to immunodeficient populations, mainly including Pneumocystis pneumonia, cytomegalovirus infection, cryptococcal meningitis, tuberculosis, nontuberculous mycobacterial disease, and bacterial pneumonia.

### Swabs collection and fungi culture

Swabs were collected from bilateral nasopharynx and oropharynx. Samples were transferred to Sabouraud broth medium (Guangzhou Detgerm Microbiological Science Ltd. Guangzhou, China) within 2 h and incubated at 25°C for vegetative growth. Examined for fungal growth daily for up to 2 weeks. The presence of white flocs or slag-like sediments was considered positive, otherwise negative. Cultures were plated onto Sabouraud dextrose agar (SDA) plates containing chloramphenicol and incubated in a constant temperature incubator at 25°C. Daily observations were made and recorded. If multiple fungal strains appeared on the plate, isolation and culture were required to obtain monoclonal strains.

### Fungal identification

Fungal identification was based on morphological and molecular methods. Fungi were identified according to their phenotypic characteristics, such as color, shape, size, sclerotia, colony surface texture, and hyphal pigmentation.

Molecular biological methods were performed when morphological methods failed to identify filamentous fungal strains. Fungal DNA was extracted using Lysis Buffer for Microorganism. The ITS (internal transcribed spacer) region of each fungus was amplified using the universal primers ITS1 and ITS4. The PCR products were electrophoresed and sent to Invitrogen (Shanghai) for sequencing in both directions. Sequence alignments were performed using NCBI BLAST.^1^ Strains showing homology of at least 97% were considered to belong to the same genus, and homology of at least 99% was considered to belong to the same species. The sequences have been submitted to NCBI Genebank database with accession number of OP103924-OP103949, and OP237033-OP237524.

Pathogenic FF included pathogenic *Aspergillus*, Mucorales (*Mucor* spp., *Rhizopus* spp., *Absidia* spp., and *Rhizomucor* spp.), and *Talaromyces marneffei*. Patients with asymptomatic pathogenic FF carriage were named pathogenic FF carriers. Pathogenic *Aspergillus* was defined as *Aspergillus* species that have been reported to cause invasive aspergillosis, including *Aspergillus fumigatus*, *Aspergillus flavus*, *Aspergillus niger*, *Aspergillus terreus*, *Aspergillus versicolor*, *Aspergillus oryzae*, and *Aspergillus sydowii* (Chiu et al., 2005; Perri et al., 2005; Schwetz et al., 2007; Cadena et al., 2021).

### Follow-up

Pathogenic FF carriers with HIV infection were followed after initial examination, including ART, plasma HIV RNA (viral load), CD4+ count, and occurrence of IFD. Patients were followed until incident IFD or the last follow-up date (December 31, 2019).

### Statistical analysis

EpiData3.1 software was used to input data, and statistical analyses were performed by SPSS 25.5 software. Controls were frequency matched to cases on sex. The chi-squared test was used for categorical variables. Logistic regression analysis was used to determine the risk factors of pathogenic FF carriage in HIV-infected individuals. Odds ratios (OR) are given with their 95% confidence intervals (95% CI). Variables with *p* < 0.10 in the univariate analysis were included in the multivariate analysis. The variance inflation factor (VIF) was used to evaluate collinearity; feature with VIF >10 was excluded. A two-tailed *p* < 0.05 was considered statistically significant.

## Results

### Baseline characteristics and fungal carriage in HIV-infected and healthy individuals

In this study, 716 HIV-infected patients and 293 healthy individuals were enrolled. No significant differences in sex and age were found between groups. Patients with HIV infection had a higher proportion of history of respiratory tract infection within 1 year (66.1% [472/716] vs. 49.5% [145/293, *p* < 0.001) than healthy individuals. There were no significant differences in environmental exposures, history of smoking or alcoholism between groups (all *p* > 0.05; Table 1). Most HIV-infected patients had received ART, and only 11% had not. Sixteen point three percent of HIV-infected patients had CD4 + T cell counts <200 cells/μl.

**Table 1:** Comparison of baseline characteristics and fungal positive rates between the HIV-infected group and the control group.

Variates	Control Group (*n* = 293)	HIV-infected Group (*n* = 716)	*p*-value
Male	246 [84.0]	602 [84.1]	0.963
Age (Years)	28 (22–45)	34 (27–42)	0.144
18–39	185 [63.1]	493 [(68.9]	–
40–63	106 [36.2]	215 [30.0]	–
>63	2 [0.7]	8 [1.1]	–
Environmental exposures within 6 months			
Rodent exposure	10 [3.4]	36 [5.0]	0.264
Hay or chaff exposure	23 [7.9]	71 [9.9]	0.305
Past medical history within 1 year			
Cured respiratory tract infections	145 [49.5]	472 [66.1]	<0.001
Cured OIs	–	128 [17.9]	–
Other infectious diseases	0 [0.0]	239 [33.4]	–
Smoking History	105 [35.8]	254 [35.5]	0.913
Alcoholism	27 [9.2]	42 [5.9]	0.056
ART	–	–	–
Non-ART	–	79 [11.0]	–
ART <1 year	–	197 [27.5]	–
ART > = 1 year	–	440 [61.5]	–
CD4 + T cell count (cells/μl)	–	385 (254–542)	–
<200	–	117 [16.3]	–
200–500	–	386 [53.9]	–
>500	–	213 [29.7]	–
HIV RNA quantitative <50 copies/ml	–	428 [89.9]^a^	–
Fungal culture results^b^			
Fungi (+)	237 [80.9]	646 [90.2]	<0.001
Yeast-like Fungi (+)	89 [30.4] ^c^	300 [41.9]^d^	0.001
Filamentous Fungi (+)	216 [73.7] ^c^	576 [80.5]^d^	0.018
Pathogenic Filamentous Fungi (+)	40 [13.7]	119 [16.6]	0.240
Pathogenic *Aspergillus* (+)	40 [13.7]	115 [16.1]	0.335
Mucorales^e^ (+)	0 [0.0]	3 [0.4]	–
*Talaromyces marneffei* (+)	0 [0.0]	2 [0.3]	–

All data are expressed as *n* [%]; *p* < 0.05 were considered statistically significant. HIV, human immunodeficiency virus; OIs, opportunistic infections; ART, antiretroviral therapy.

a Information about HIV RNA quantitative was missing for 240 HIV-infected patients.

b Upper respiratory tract samples include nasopharyngeal swabs and oropharyngeal swabs.

c Sixty-eight of them carried both yeast-like fungi and filamentous fungi.

d Two hundred and thirty of them carried both yeast-like fungi and filamentous fungi.

e Mucorales that cause mucormycosis.

**Figure 1:**
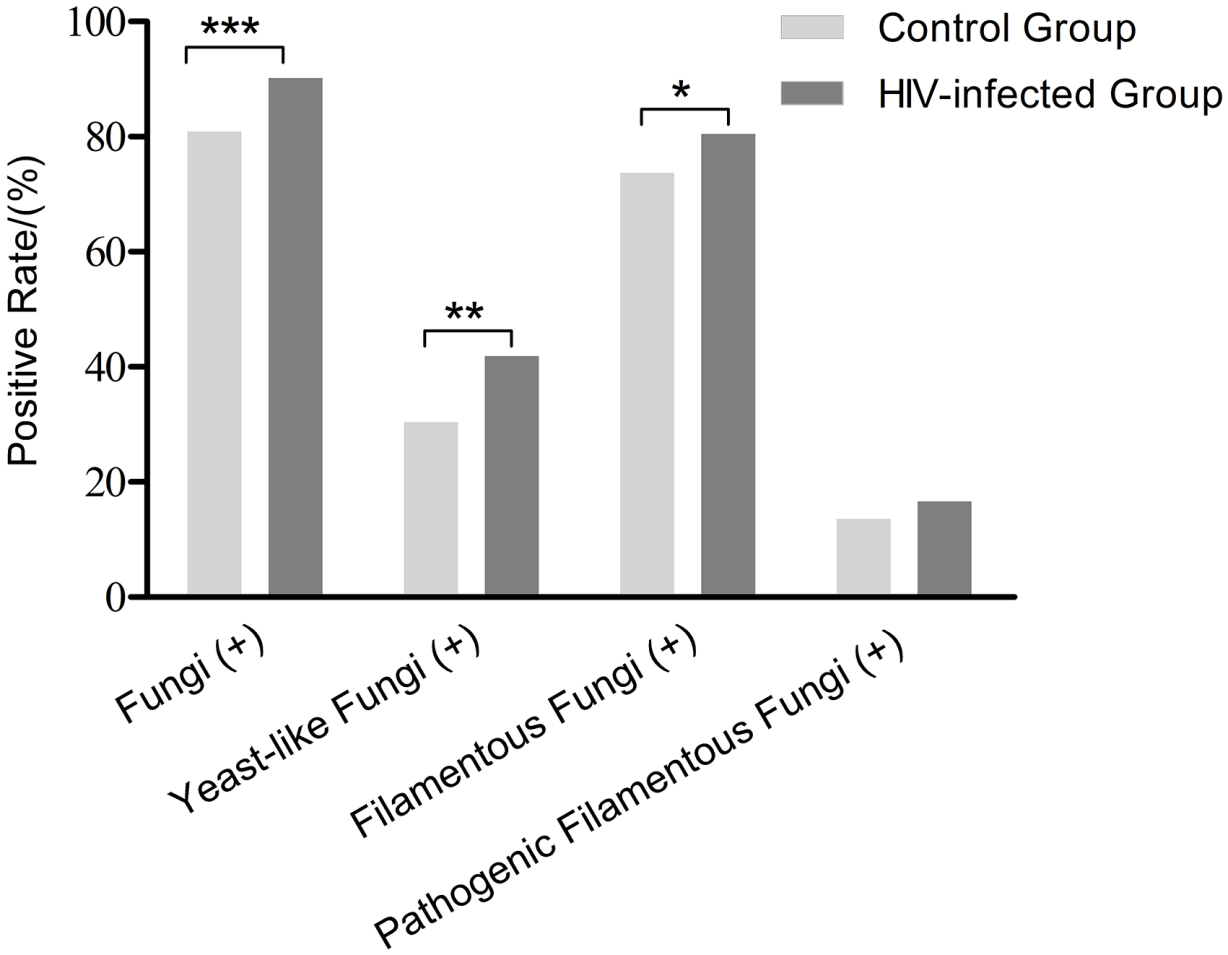
Positive rates of fungal carriage in the HIV-infected group and the control group. *p < 0.05, **p < 0.01, ***p < 0.001.

All patients underwent oropharyngeal and nasopharyngeal swabs for fungi examinations. In the HIV-infected group, fungi were isolated in 90.2% [646 /716] of patients. Three hundred (41.9%) cases had yeast-like fungi and 576 (80.5%) had FF, higher than those in the control group (all *p* < 0.05; Figure 1). The positive rate of pathogenic FF in the HIV-infected group was similar to that in the control group (16.6% [119 /716] vs. 13.7% [40 /293], *p* = 0.24; Table 1). Mucorales were found in 3 HIV-infected individuals and *Talaromyces marneffei* in 2 HIV-infected individuals, but not in healthy controls.

The identification results of FF were listed in Supplementary Table S1. We identified 623 FF strains in the HIV-infected group and 235 FF strains in the control group, respectively. In HIV-infected group, 125 pathogenic FF strains were identified, including 24.0% [30/125] of *Aspergillus fumigatus*, 14.4% [18/125] of *Aspergillus flavus*, 26.4% [33/125] of *Aspergillus niger*, 4.0% [5/125] of *Aspergillus terreus*, 11.2% [14/125] of *Aspergillus versicolor*, 4.8% [6/125] of *Aspergillus oryzae*, 11.2% [14/125] of *Aspergillus Sydowii,* 1.6% [2/125] of *Talaromyces marneffei*, 0.8% [1/125] of *Rhizomucor variabilis,* 0.8% [1/125] of *Rhizopus microsporus*, and 0.8% [1/125] (Table 2).

**Table 2:** Identification results of pathogenic filamentous fungi in HIV-infected individuals and healthy controls.

Species	Number of Strains	
	HIV-infected group	Control group
*Aspergillus fumigatus*	30 [24.0]	11 [26.2]
*Aspergillus flavus*	18 [14.4]	7 [16.7]
*Aspergillus niger*	33 [26.4]	9 [21.4]
*Aspergillus terreus*	5 [4.0]	4 [9.5]
*Aspergillus versicolor*	14 [11.2]	6 [14.3]
*Aspergillus oryzae*	6 [4.8]	1 [2.4]
*Aspergillus sydowii*	14 [11.2]	4 [9.5]
*Talaromyces marneffei*	2 [1.6]	0 [0.0]
*Rhizomucor variabilis*	1 [0.8]	0 [0.0]
*Rhizopus microsporus*	1 [0.8]	0 [0.0]
*Rhizopus sp.*	1 [0.8]	0 [0.0]
Total	125 [100.0]	42 [100.0]

All data are expressed as *n* [%].

### Risk factors of pathogenic FF carriage in HIV-infected individuals

Among HIV-infected individuals, proportions of cured OIs history (27.7% [33/119] vs. 15.9% [95/597], *p* = 0.002), and smoking history (44.5% [53/119] vs. 33.7% [201/597], *p* = 0.024) were significantly higher in pathogenic FF carriers than those of non-carriers (Table 3). In univariate analysis, OIs, and smoking history showed strong associations with pathogenic FF carriage in HIV-infected individuals (*p* < 0.05; Table 3). In multivariate analysis, after adjusting for the influence of rodent exposure, hay or chaff exposure, and respiratory tract infections, OIs (OR, 1.97; 95% CI, 1.23–3.13, *p* = 0.004), and smoking history (OR, 1.55; 95%CI, 1.03–2.32, *p* = 0.035) were independent risk factors of pathogenic FF carriage in HIV-infected individuals (Table 4).

**Table 3:** Univariate analysis of risk factors for pathogenic filamentous fungi carriage in HIV-infected group.

Variates	Pathogenic filamentous fungi^a^		OR (95% CI)	*p*-value
	Non-carrier (*n* = 597)	Carrier (*n* = 119)		
Male	500 [83.8]	102 [85.7]	1.16 (0.67–2.03)	0.593
Age groups	–	–	–	0.627
18–39 years	415 [69.5]	78 [65.5]	1 (ref)	–
40–63 years	175 [29.3]	40 [33.6]	1.22 (0.80–1.85)	–
>63 years	7 [1.2]	1 [0.8]	0.76 (0.09–6.26)	–
Environmental exposures within 6 months				
Rodent exposure	26 [4.4]	10 [8.4]	2.02 (0.95–4.30)	0.070
Hay or chaff exposure	54 [9.0]	17 [14.3]	1.68 (0.93–3.01)	0.083
Cured infection within 1 year				
Respiratory tract infections	402 [67.3]	70 [58.8]	0.69 (0.46–1.04)	0.075
Other infectious diseases	201 [33.7]	38 [31.9]	0.92 (0.61–1.41)	0.714
OIs	95 [15.9]	33 [27.7]	2.03 (1.28–3.20)	0.002
Smoking history	201 [33.7]	53 [44.5]	1.58 (1.06–2.36)	0.024
Alcoholism	32 [5.4]	10 [8.4]	1.62 (0.77–3.39)	0.201
ART	–	–	–	0.165
Non-ART	71 [11.9]	8 [6.7]	1 (ref)	–
ART <1 year	167 [28.0]	30 [25.2]	1.59 (0.70–3.65)	–
ART ≥1 year	359 [60.1]	81 [68.1]	2.00 (0.93–4.32)	–
CD4 + T cell count	–	–	–	0.570
<200 cell/μl	95 [15.9]	22 [18.5]	1 (ref)	–
200–500 cells/μl	327 [54.8]	59[49.6]	0.78 (0.45–1.34)	–
>500 cells/μl	175 [29.3]	38 [31.9]	0.94 (0.52–1.68)	–
HIV RNA quantitative <50 copies/ml	347 [89.0] ^b^	81 [94.2] ^c^	0.50 (0.19–1.30)	0.154

Data were expressed as n [%], or odds ratios (OR) and 95% confidence interval (CI), as appropriate. HIV, human immunodeficiency virus; OR, odds ratio; OIs, opportunistic infections; ART, antiretroviral therapy.

a Upper respiratory tract samples include nasopharyngeal swabs and oropharyngeal swabs.

b Information about HIV RNA quantitative was missing for 207 non-carriers.

c Information about HIV RNA quantitative was missing for 33 carriers; All variables of variance inflation factor (VIF: 1.023–2.683) were less than 5–10; Variables with *p* < 0.10 in the univariate analysis were included in the multivariate analysis.

**Table 4:** Multivariate analysis of risk factors for pathogenic filamentous fungi carriage in HIV-infected group.

Variates	OR	95% CI		*p*-value
		Lower	Upper	
Rodent exposure	2.08	0.95	4.57	0.068
Hay or chaff exposure	1.56	0.85	2.87	0.154
Respiratory tract infections	0.67	0.44	1.01	0.056
Smoking history	1.55	1.03	2.32	0.035
OIs	1.97	1.23	3.13	0.004
Constant	0.17	–	–	<0.001

HIV, human immunodeficiency virus; OR, odds ratio; OIs, opportunistic infections.

### IFD in pathogenic FF carriers with HIV infection during follow-up

In this study, all 119 pathogenic FF carriers with HIV infection were followed through the end of 2019, and none developed IFD. As shown in Table 5, 93.3% of the patients have received ART at baseline, and 100% during follow-up. During follow-up, 90% of the patients had CD4+ count >200 cells/μl, 95.4% had sustained virologic suppression (HIV RNA quantitative <50 copies/ml; Table 5).

**Table 5:** Baseline characteristics and follow-up of pathogenic filamentous fungi carriers with HIV infection.

	Baseline (*n* = 119)	Follow-up (*n* = 119)
CD4 + T cell count		
<50 cells/μl	3 [2.5]	2 [1.7]
≥50, <200 cells/μl	19 [16.0]	10 [8.4]
200–500 cells/μl	59 [49.6]	56 [47.1]
> 500 cells/μl	38 [31.9]	51 [42.9]
HIV RNA quantitative <50 copies/ml^a^	81 [94.2]	104 [95.4]
ART (n [%])	111 [93.3]	119 [100.0]
Duration of ART (Months)	25 (11–46)	52 (35–73)
Minimum duration of ART (Months)	0	28
Longest duration of ART (Months)	135	163
Invasive fungal infection (n [%])	0 [0.0]	0 [0.0]

Data are expressed as medians (interquartile range), or n [%], as appropriate. HIV, human immunodeficiency virus; ART, antiretroviral therapy.

a Information about HIV RNA quantitative was missing for 33 patients at baseline, and 10 patients at follow-up.

## Discussion

In the present study, we found that asymptomatic fungal carriage in the upper respiratory tract is common in HIV-infected individuals, and both smoking and cured OIs history significantly increase their risk of pathogenic FF carriage. Besides, asymptomatic carriage of pathogenic FF in HIV-infected individuals does not appear to increase the risk of IFD.

As the access to ART expands, AIDS-relative mortality rates declined year by year. However, IFD remains a fatal threat to patients with advanced AIDS, and its mortality is second only to that of tuberculosis (Hoving et al., 2020). Most occurring cases of IFD begin by inhalation of fungal spores or the colonization of fungi (Von Lilienfeld-Toal et al., 2019; Pathakumari et al., 2020). With the destruction of immunity, FF carried on the mucosal surface can break through the mucosal barrier and cause IFD.

The upper respiratory tract is a major portal for microbial invasion, but few studies had investigated fungal carriage of the upper respiratory tract in patients with HIV infection. We reported the carriage of fungi in HIV-infected and healthy individuals and found that the asymptomatic fungal carrier rate was up to 90.2% in HIV-infected individuals and 80.9% in healthy controls. In our study, 41.9% of the HIV-infected individuals carried yeast-like fungi, which is consistent with previous studies (Maurya et al., 2013; Goulart et al., 2018; Aboualigalehdari et al., 2020). Although FF carrier rate was higher in HIV-infected individuals than that in healthy persons in our report, it was mainly caused by non-pathogenic FF. Pathogenic *Aspergillus* was the most common pathogenic FF, which was present in 16.1% of HIV-infected individuals. The prevalence of pathogenic *Aspergillus* carriers was similar between patients with and without HIV infection, which could be interpreted by host immune response. Human immunity against fungal hyphae is mainly performed by phagocytic cells, especially neutrophils (Rex et al., 1990; Mircescu et al., 2009). The phagocytic cell functions are intact in HIV-infected individuals as HIV primarily infects CD4+ T cells (Singh, 2021). Besides, the majority of participants in our study had relatively preserved immune function with CD4+ counts of more than 200 cells/μl. We reported three asymptomatic Mucorales carriers and two *Talaromyces marneffei* carriers in the HIV-infected group. This suggests that HIV-infected individuals are still at risk of being colonized by the above fungi even if their immune function is relatively preserved. Notably, patients with advanced AIDS are frequently comorbid with neutropenia, and the colonization of pathogenic FF may be a potential threat to IFD (Levine et al., 2006; Shi et al., 2014).

In this study, HIV-infected individuals with smoking history were found to have a 55% increased risk of pathogenic FF carriage (OR, 1.55; 95%CI, 1.03–2.32, *p* = 0.035). Tobacco smoke exerts widespread toxic effects on the immune system (Yamaguchi, 2019). It can inhibit phagocytosis and killing of foreign bodies by macrophages and neutrophils in the mucosa, inhibit the expression of interferon gamma (IFN-γ) gene, and increase regulatory T lymphocyte (Treg) counts, thus impairing the capacity of the body to remove fungi at mucosal surfaces (Yamaguchi, 2019). A study in the United States also found that smoking increased the risk of *Pneumocystis* colonization among HIV-infected men (Morris et al., 2004). Smoking is common among HIV-infected individuals and is associated with adverse outcomes (Helleberg et al., 2013, 2015; Giles et al., 2018). It is necessary to promote health education to reduce the smoking rate of HIV-infected people. The history of cured OIs in HIV-infected individuals was also found to be a risk factor for pathogenic FF carriage, which may be related to low immunity. The occurrence of OIs usually means severe immunodeficiency in patients with HIV infection, and although most patients achieve persistent virological suppression after ART, there is usually incomplete immune reconstitution, resulting in inadequate clearance of mucosal fungi (Yang et al., 2020).

In this study, pathogenic FF carriers with HIV infection were followed up for about 2.5 years, and none developed IFD. All of those patients received ART for at least 28 months, and most achieved sustained virologic suppression and immune reconstitution. This suggests that effective ART is critical for the prevention of IFD in HIV-infected individuals. Pathogenic FF carriage does not increase the risk of IFD in patients with good immune-controlled HIV infection.

This is the first study to investigate asymptomatic fungal carriers among HIV-infected individuals and healthy persons. Previous studies concentrate more on the colonization of bacteria and candida, and the prevalence and risk factors of FF colonization in HIV-infected patients were unknown before this study (Maurya et al., 2013; Goulart et al., 2018; Rossetti et al., 2019; Aboualigalehdari et al., 2020).

Our study had some limitations. Firstly, the definition of pathogenic FF was based on what was reported in the literature, other FF may be pathogenic but have been missed. Secondly, molecular methods were not used in all isolates, but only when identification by morphological methods was not possible. Despite careful identification, some fungal strains without molecular biological identification may have been misidentified. Therefore, further study with complete molecular methods is needed to confirm our findings. Thirdly, most of the participants had received ART and achieved immune reconstitution, so the conclusions from this study may only apply to those who had received effected ART and achieved sustained virologic suppression and immune reconstitution. Notably, once ART is interrupted and the immune deficiency is aggravated, the colonized fungi may invade the host and develop IFD. Further study is needed to explore the mechanism of fungal colonization, the relationship between colonization and infection, and the way to improve the micro-ecological environment in patients with HIV infection.

In summary, asymptomatic fungal carriage is common in HIV-infected individuals but may not develop IFD in those who achieved immune reconstitution. Smoking and cured OIs history increase the risk of pathogenic FF carriage. Smoking abstinence and ART adherence are especially important for these patients.

## Data availability statement

The datasets presented in this study can be found in online repositories. The names of the repository/repositories and accession number(s) can be found below: NCBI GenBank database with accession number of OP103924-OP103949 and OP237033-OP237524.

## Ethics statement

The studies involving human participants were reviewed and approved by Medical Ethics Committee of the Guangzhou Eighth People’s Hospital. The patients/participants provided their written informed consent to participate in this study.

## Author contributions

XC: conceptualization (equal), formal analysis (lead), and writing – original draft preparation (lead). YC: conceptualization (equal), methodology (lead), and writing – review and editing (equal). MC: conceptualization (equal), investigation (lead), and writing – review and editing (equal). HW: investigation (equal), methodology (equal), and writing – review and editing (equal). PD, HL, HZ, QL, SZ, and WCh: investigation (equal) and methodology (equal). ZY: methodology (equal) and formal analysis (equal). WCa: supervision (lead) and writing – review and editing (equal). XT and LL: conceptualization (lead), project administration (lead), and writing – review and editing (lead). All authors contributed to the article and approved the submitted version.

## Funding

This work was supported by grants from the Guangzhou Basic Research Program on People’s Livelihood Science and Technology (grant number: 202002020005) and the National Natural Science Foundation of China (grant number: 82072265).

## Conflict of interest

The authors declare that the research was conducted in the absence of any commercial or financial relationships that could be construed as a potential conflict of interest.

## Publisher’s note

All claims expressed in this article are solely those of the authors and do not necessarily represent those of their affiliated organizations, or those of the publisher, the editors and the reviewers. Any product that may be evaluated in this article, or claim that may be made by its manufacturer, is not guaranteed or endorsed by the publisher.

## Footnotes

^1^ https://blast.ncbi.nlm.nih.gov/
