# Supplementary material for: HIV-infected patients rarely develop invasive fungal diseases under good immune reconstitution after ART regardless high prevalence of pathogenic filamentous fungi carriage in nasopharynx/oropharynx
Source: Front Microbiol. 2022 Nov 2;13:968532. doi: 10.3389/fmicb.2022.968532 (PMC9666755; doi:10.3389/fmicb.2022.968532)
Supplement: Supplementary file 1 [file Table_1.DOCX]

Supplementary Material

Supplemental Table S1 Identification results of filamentous fungi in HIV-infected individuals and healthy controls

| **Genus** | **Species** | **Number of Strains** | |
| --- | --- | --- | --- |
|  |  | **HIV-infected Group** | **Control Group** |
| *Aspergillus* | *A. fumigatus* | 30 | 11 |
|  | *A. flavus* | 18 | 7 |
|  | *A. niger* | 33 | 9 |
|  | *A. terreus* | 5 | 4 |
|  | *A. versicolor* | 14 | 6 |
|  | *A. oryzae* | 6 | 1 |
|  | *A. sydowii* | 14 | 4 |
|  | *A. aculeatus* | 26 | 10 |
|  | *A. japonicus* | 5 | 2 |
|  | *A. sclerotiorum* | 4 | 0 |
|  | *A. ochraceus* | 3 | 2 |
|  | *A. elegans* | 2 | 1 |
|  | *A. nomius* | 1 | 2 |
|  | *A. puniceus* | 1 | 0 |
|  | *A. stellatus* | 1 | 0 |
|  | *A. subramanianii* | 1 | 1 |
|  | *A. tabacinus* | 1 | 0 |
|  | *A. westerdijkiae* | 11 | 0 |
|  | *A. clavatus* | 1 | 1 |
|  | *A. amstelodami* | 0 | 3 |
|  | *A. caesiellus* | 1 | 0 |
|  | *A. tubingensis* | 0 | 1 |
|  | *Aspergillus sp.* | 4 | 2 |
| *Penicillium* | *P. citrinum* | 76 | 15 |
|  | *P. oxalicum* | 8 | 4 |
|  | *P. commune* | 5 | 0 |
|  | *P. rolfsii* | 4 | 0 |
|  | *P. steckii* | 4 | 0 |
|  | *P. griseofulvum* | 1 | 1 |
|  | *P. infrabuccalum* | 3 | 0 |
|  | *P. mallochii* | 2 | 1 |
|  | *P. aurantiocandidum* | 1 | 0 |
|  | *P. chrysogenum* | 3 | 0 |
|  | *P. pedernalense* | 1 | 0 |
|  | *P. sclerotiorum* | 3 | 4 |
|  | *P. sumatraense* | 1 | 0 |
|  | *P. thomii* | 1 | 0 |
|  | *P. chermesinum* | 0 | 2 |
|  | *P. coffeae* | 1 | 1 |
|  | *P. fellutanum* | 2 | 2 |
|  | *Penicillium sp.* | 6 | 1 |
| *Cladosporium* | *C. cladosporioides* | 4 | 2 |
|  | *C. halotolerans* | 10 | 0 |
|  | *C. oxysporum* | 3 | 0 |
|  | *C. tenuissimum* | 2 | 2 |
|  | *C. parahalotolerans* | 4 | 0 |
| *Talaromyces* | *T. marneffei* | 2 | 0 |
|  | *T. verruculosus* | 9 | 0 |
|  | *T. purpureogenus* | 4 | 0 |
|  | *T. aurantiacus* | 2 | 0 |
|  | *T. amestolkiae* | 1 | 0 |
|  | *T. cnidii* | 2 | 0 |
|  | *T. thailandensis* | 0 | 1 |
|  | *T. dimorphus* | 1 | 0 |
| Others | *Rhizomucor variabilis* | 1 | 0 |
|  | *Rhizopus microsporus* | 1 | 0 |
|  | *Rhizopus sp.* | 1 | 0 |
|  | *Eutypella sp.* | 78 | 53 |
|  | *Eutypa linearis* | 1 | 0 |
|  | *Eutypella scoparia* | 2 | 1 |
|  | *Phomopsis sp.* | 5 | 0 |
|  | *Coprinellus radians* | 8 | 0 |
|  | *Diaporthe phaseolorum* | 5 | 3 |
|  | *Diaporthe phaseolorum var.* | 1 | 0 |
|  | *Annulohypoxylon nitens* | 6 | 1 |
|  | *Daldinia eschscholtzii* | 18 | 11 |
|  | *Diaporthe sp.* | 5 | 0 |
|  | *Fusarium fujikuroi* | 4 | 0 |
|  | *Colletotrichum gloeosporioides* | 3 | 0 |
|  | *Microsphaeropsis arundinis* | 4 | 0 |
|  | *Paecilomyces lilacinus* | 2 | 0 |
|  | *Phomopsis heveicola* | 2 | 0 |
|  | *Sordariomycetes sp.* | 2 | 0 |
|  | *Trichoderma citrinoviride* | 6 | 2 |
|  | *Trichoderma harzianum* | 6 | 3 |
|  | *Acrocalymma medicaginis* | 2 | 0 |
|  | *Aporospora sp.* | 1 | 0 |
|  | *Arthrinium arundinis* | 1 | 1 |
|  | *Arthrinium rasikravindrae* | 1 | 0 |
|  | *Ascomycota sp.* | 1 | 0 |
|  | *Basidiomycota sp.* | 1 | 0 |
|  | *Botryosphaeria dothidea* | 4 | 0 |
|  | *Cladobotryum asterophorum* | 5 | 2 |
|  | *Clonostachys epichloe* | 1 | 0 |
|  | *Colletotrichum tropicicola* | 1 | 0 |
|  | *Colletotrichum boninense* | 1 | 0 |
|  | *Colletotrichum taiwanense* | 1 | 0 |
|  | *Coniothyrium sp.* | 1 | 0 |
|  | *Cucurbitariaceae sp.* | 7 | 3 |
|  | *Curvularia geniculata* | 1 | 0 |
|  | *Deconica coprophila* | 1 | 0 |
|  | *Diaporthe eucalyptorum* | 1 | 0 |
|  | *Diaporthe hongkongensis* | 1 | 0 |
|  | *Diaporthe longicolla* | 2 | 0 |
|  | *Fusarium proliferatum* | 2 | 0 |
|  | *Fusarium solani* | 2 | 1 |
|  | *Gibberella moniliformis* | 1 | 0 |
|  | *Hypocreales sp*. | 4 | 2 |
|  | *Hypocrea lactea* | 0 | 1 |
|  | *Hypoxylon monticulosum* | 3 | 3 |
|  | *Hypoxylon sp.* | 1 | 1 |
|  | *Hypoxylon investiens* | 6 | 9 |
|  | *Leptospora rubella* | 3 | 0 |
|  | *Leptospora sp.* | 1 | 0 |
|  | *Microascales sp.* | 1 | 0 |
|  | *Ochroconis mirabilis* | 1 | 0 |
|  | *Ophiosphaerella agrostidis* | 1 | 0 |
|  | *Peniophora lycii* | 2 | 2 |
|  | *Periconia thailandica* | 1 | 0 |
|  | *Peroneutypa scoparia* | 1 | 0 |
|  | *Phaeosphaeriopsis musae* | 1 | 0 |
|  | *Phaeosphaeriopsis sp.* | 1 | 0 |
|  | *Phanerochaete chrysosporium* | 5 | 0 |
|  | *Phanerochaete sp.* | 3 | 0 |
|  | *Phomopsis liquidambari* | 1 | 0 |
|  | *Pleurotus nebrodensis* | 1 | 0 |
|  | *Pseudopithomyces maydicus* | 1 | 1 |
|  | *Purpureocillium lilacinum* | 4 | 1 |
|  | *Pestalotiopsis microspora* | 0 | 1 |
|  | *Plectosphaerella cucumerina* | 0 | 2 |
|  | *Rhytidhysteron rufulum* | 3 | 0 |
|  | *Schizophyllum commune* | 1 | 1 |
|  | *Trametes polyzona* | 2 | 0 |
|  | *Whalleya microplaca* | 1 | 0 |
|  | *Xenoacremonium recifei* | 4 | 1 |
|  | *Annulohypoxylon stygium* | 4 | 6 |
|  | *Arthrinium sp.* | 1 | 2 |
|  | *Fusarium chlamydosporum* | 1 | 2 |
|  | *Fusarium oxysporum* | 13 | 7 |
|  | *Fusarium striatum* | 3 | 5 |
|  | *Neofusicoccum parvum* | 0 | 1 |
|  | *Pleosporales sp.* | 0 | 2 |
|  | *Roussoella solani* | 0 | 1 |
| Total |  | 623 | 235 |
